# Metachronous Second Primary in the Form of Nasopharyngeal Carcinoma Following Treatment of Small Cell Neuroendocrine Carcinoma of the Head and Neck: Dual Tracer PET/CT Findings Highlighting SSTR2 Expression and Its Theranostic Implications

**DOI:** 10.1055/s-0044-1790599

**Published:** 2024-09-30

**Authors:** Yeshwanth Edamadaka, Sunita Nitin Sonavane, Sandip Basu

**Affiliations:** 1 Radiation Medicine Centre, Bhabha Atomic Research Centre, Tata Memorial Hospital Annexe, Jerbai Wadia Road, Parel, Mumbai, India; 2 Homi Bhabha National Institute, Mumbai, India

**Keywords:** sinonasal carcinoma, small cell neuroendocrine carcinoma, NEC, ^18^ F-FDG, [ ^68^ Ga]Ga-DOTATATE, nasopharyngeal carcinoma, metachronous second primary malignancy

## Abstract

Patients of head and neck squamous cell carcinoma (HNSCC) experience increased risk of developing second primary cancer (SPC) necessitating active surveillance during their disease course. SPCs are associated with poor prognosis and are the leading cause of long-term morbidity and mortality impacting survival of patients with HNSCC. Small cell neuroendocrine carcinoma (SmNEC) is a rare but aggressive neoplasm with poor prognosis and high risk of local recurrence and distant metastasis. We report an exceedingly rare case of nasopharyngeal carcinoma (NPC) presenting as a recurrence in the form of metachronous second primary to primary SmNEC 9 years after chemotherapy. The dual tracer positron emission tomography and computed tomography (PET/CT) imaging approach ([ ^68^ Ga]Ga-DOTATATE-PET/CT with ^18^ F-FDG-PET/CT) was explored in such metachronous NPCs, and the findings are illustrated with its potential for theranostic applications. NPC is a rare malignancy with significant geographical variations in incidence rates. Somatostatin receptor 2 (SSTR2) expression in NPC is well documented and can serve as a potential theragnostic marker in advanced NPC where the successful outcome is minimal with currently available treatment modalities.

## Introduction

Head and neck squamous cell carcinoma (HNSCC) shows increased risk of developing second primary cancer (SPC). Patients with HNSCC have an approximately yearly incidence of 3 to 7% of SPC with a 20-year cumulative risk of 36%. 1 SPC is associated with three times higher risk of mortality than metastatic disease and accounts for one-third of HNSCC deaths. 2 The incidence of SPC in a large population cohort was found to be 12.3%. Excess SPC risk was found to be standardized incidence ratios (SIRs) of 2.07 and absolute excess absolute risk (EAR) of 164.3 cases per 10,000 person-years at risk. 3 We present a case of small cell neuroendocrine carcinoma (SmNEC) treated on chemotherapy alone, which showed significant clinical and imaging response. However, clinical evidence of recurrence was observed 9 years later, and the final histological diagnosis was nasopharyngeal carcinoma (NPC).

## Case History

A 30-year-old male patient presented with recurrent epistaxis, headache, and swelling on the right side of the face for 3 months with progressive epiphora in the right eye. He was a known smoker for 15 years and tobacco chewer for 5 years. On clinical examination, a mass was seen occupying the right nostril with adjacent fullness in the cheek, right-sided proptosis with normal vision, and no enlarged neck nodes. Magnetic resonance imaging (MRI) evaluation showed right nasal cavity mass causing buckling of the lateral nasal wall with remodeling of the right inferior orbital wall and associated minimal displacement of the right eye suspicious for malignancy. Fluorine-18 fluorodeoxyglucose positron emission tomography and computed tomography ( ^18^ F-FDG-PET/CT) scan done for staging revealed a right nasal cavity mass measuring 8.7 × 4.7 × 6 cm, maximum standardized uptake value (SUVmax) of 6.3 with locoregional extension into the right maxillary sinus and the nasopharynx. There was evidence of destruction of the cribriform plate of the right ethmoid sinus with the mass reaching up to the skull base. The right nasal mass biopsy showed a small round cell tumor with crushing artifacts and areas of necrosis. On immunohistochemistry (IHC), the tumor cells were positive for AE1/AE3 and CD56 and negative for LCA, Mic-2, desmin, synaptophysin, and chromogranin with Mib-1 labeling index of 50 to 55% establishing a diagnosis of SmNEC. He then underwent neoadjuvant chemotherapy with cisplatin and etoposide. After the first cycle, there was significant response with decreased headache, epistaxis, epiphora, and complete relief of nasal blockade. Postchemotherapy hematology workup showed a grade III neutropenia and his chemotherapy was delayed. He received his second chemotherapy after recovery. His computed tomography (CT) scan showed no disease, and he was unwilling to undergo any local treatment. He was asymptomatic when evaluated 6 months following the two cycles of chemotherapy and no other treatment. He did not follow up despite multiple efforts explaining the risk of recurrence by the attending physician and presented with similar symptoms as 9 years earlier including epistaxis and breathing difficulty, which were progressive in nature.

**Fig. 1:**
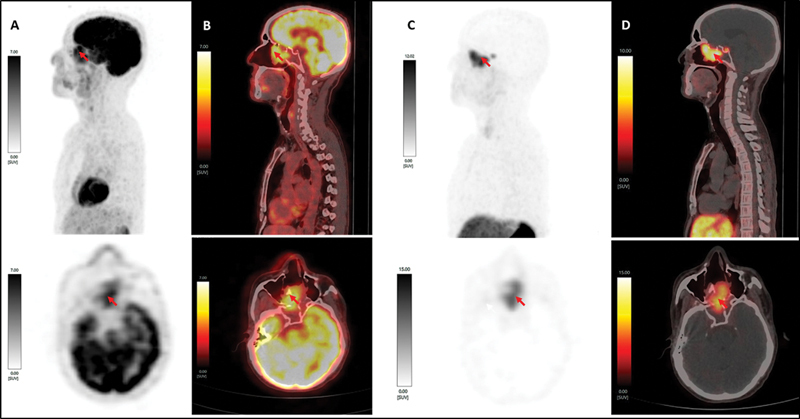
Dual tracer PET/CT ( ^18^ F-FDG and [ ^68^ Ga]Ga-DOTATATE) illustrative images showing nasopharyngeal carcinoma (NPC) measuring 4.1 × 1.8 × 4 cm ( *red arrow* ). **(A)** Left lateral maximum-intensity projection (MIP) of ^18^ F-FDG-PET scan (upper image) showing uptake in the posterior half of the nasal cavity (maximum standardized uptake value [SUVmax] of 6.59) and ^18^ F-FDG-PET axial image (lower image) showing a well-delineated mass from physiological brain uptake. **(B)** Sagittal fused ^18^ F-FDG-PET/CT (upper image) showing mass reaching up to the cribriform plate superiorly and posteriorly causing osteolysis with protrusion into the sphenoid sinus and the axial fused image (lower image) showing involvement of the bony nasal septum with SUVmax 7.04. **(C)** Left lateral MIP of [ ^68^ Ga]Ga-DOTATATE-PET (upper image) and axial image (lower image) show increased somatostatin receptor (SSTR) expression in a well-delineated mass with good lesion to background contrast. **(D)** Sagittal fused [ ^68^ Ga]Ga-DOTATATE-PET/CT (upper image) and axial fused image (lower image) showing heterogenous SSTR expression with SUVmax 10.17.

He underwent a contrast-enhancing CT scan, which showed a solid enhancing mass in the posterior half of the nasal cavity with local infiltration into the sphenoid sinus and osteolysis of the bony nasal septum. An illustrative image showing a dual tracer, that is, ^18^ F-FDG and [ ^68^ Ga]Ga-DOTA-Tyr3-octreotate ([ ^68^ Ga]-Ga-DOTATATE) PET/CT demonstrating a suspicious mass in the nasal cavity is shown in Fig. 1 . Repeat biopsy showed keratinizing stratified squamous epithelium with individual cells having hyperchromatic round nucleus and moderate amount of eosinophilic cytoplasm features consistent with NPC. He underwent five cycles of injection carboplatin and injection paclitaxel every 3 weeks. Clinically, his symptoms improved with reduction in size of the NPC, 9 months from the diagnosis.

## Discussion

An SPC is defined as the subsequent primary cancer occurring at least 2 months after the first cancer diagnosis, tumors arising in different sites or at the same site with different histological findings. 4 SPCs are associated with poor prognosis and are the leading cause of long-term morbidity and mortality, impacting survival of patients. Field cancerization effect describes the association of HNSCC with SPC in which carcinogenic effects from tobacco and alcohol may act simultaneously on the aerodigestive tract, elevating the risk of cancer through the head and neck, lung, and esophagus. 5 The risk of HNSCC attributable to alcohol and tobacco exposure varies by subsite; a case control study showed that tobacco is strongly associated with risk of laryngeal cancers, while alcohol is associated with oropharyngeal cancers. 6

SmNEC of the nasal cavity and paranasal sinuses is a rare but aggressive neoplasm with poor prognosis and high risk of local recurrence and distant metastasis. Extrapulmonary SmNEC accounts for 2.5 to 5% of all SmNECs. 7 The most commonly affected sites in the head and neck are the larynx, oral cavity, and pharynx, with SNEC of the nasal cavity and paranasal sinuses constituting an even smaller proportion. 7 A systemic review of 80 cases of sinonasal SmNEC reported nasal cavity as the most common site of involvement and the majority of cases presented with the American Joint Committee on Cancer (AJCC) stage IV disease. The median time for recurrence was 9 months in a total of 32 patients who had a local, regional, or distant metastasis. 8 The histopathology of sinonasal SmNEC mirrors the pulmonary equivalent with dense cellular tumors arranged in sheets, cords, and ribbons with cells having high nuclear-to-cytoplasmic ratios. Conclusive diagnosis of SmNEC is based on IHC, such as positive CD56 and cytokeratin markers; positive synaptophysin or chromogranin A expression is not necessary for diagnosis. The MiB-1 index represents the cell proliferation activity of SmNEC. 9 The treatment response depends on the stage, differentiation grade, and cell proliferation rate rather than by the TNM ( *t* umor size, *n* ode involvement, and *m* etastasis status) classification. 10 ^18^ F-FDG-PET/CT metabolic parameters help in characterization of some of the common sinonasal neoplasms into different histopathological subgroups, but there is a considerable overlap. 11 Liu et al 12 demonstrated significant somatostatin receptor (SSTR) expression in a metastatic high-grade SmNEC in nasal cavity and vertebral body lesions, which were not detected on CT scan. Adnan and Basu 13 reported a case of recurrent metastatic SmNEC. Due to limited options and significant SSTR and FDG expression, a combined protocol of [ ^177^ Lu]Lu-DOTATATE and platinum-based chemotherapy was found to be an management effective.

There have been a couple of case reports on the incidence of SmNEC after successful treatment of NPC, which was diagnosed on histopathology. 14 15 On the contrary, we present a case of NPC presenting as a recurrence to SmNEC treated with chemotherapy alone.

NPC is a rare malignancy with significant geographical variations in incidence rates. We report a case of NPC as an SPC in an already treated case of SmNEC, which to our knowledge has not been reported yet. 16 ^18^ F-FDG-PET/CT did not provide any survival benefit in stage I and II NPC patients However, ^18^ F-FDG can provide accurate diagnosis of lymph nodes compared to conventional modalities including MRI/CT in NPC. 17 It is highly recommended for patients with bilateral lymphadenopathy or palpable lymph nodes below cricoid cartilage, due to high risk of occult distant metastasis. ^18^ F-FDG-PET/CT helps in radiotherapy planning, prognostic information, assessment of therapeutic response, and follow-up for detection of recurrence. 18

Somatostatin receptor 2 (SSTR2) expression has been documented in NPC in case reports. 19 LMP1, a protein product of Epstein–Barr virus (EBV), governs proliferative signaling pathways associated with epidermal growth factor receptor (EGFR) and nuclear factor kappa B (NF-κB). NF-κB signaling has been demonstrated to regulate SSTR2 expression in NPC. 20 Xu et al 21 showed a positive correlation between EGFR and SSTR2, associated with higher risk of progression and worse outcome. EGFR-targeted drugs have been shown to be effective in recent studies, but drug resistance poses a significant challenge. SSTR2 expression in NPC can be a potential theragnostic biomarker. Zhao et al 22 reported comparable diagnostic efficiency of [ ^68^ Ga]Ga-DOTATATE-PET/CT with ^18^ F-FDG-PET/CT, with better image contrast. Recently Zheng et al 23 reported [ ^68^ Ga]Ga-DOTATATE to be superior to [ ^68^ Ga]Ga-FAPI PET/CT in visualizing the primary cancer and in detecting local recurrence and metastatic lesions in NPC. Intensity-modulated radiotherapy (IMRT) is the mainstay of treatment with good outcome, and optimal treatment strategy for patients with advanced NPC is not standardized. 24 Dual-tracer PET/CT functional imaging can biologically characterize tumors so that their treatments can be optimized by assessing tumor biology on a continuous scale and evaluating intra- and inter-lesional heterogeneity. 25

## Conclusion

We conclude that the relationship between SmNEC and NPC is unusual and can be attributed to shared risk factors. Further, somatostatin-based molecular imaging can serve as a feasible option in the management of recurrence or metastatic NPC.
